# The Sources of Physical Science; Being an Introduction to the Study of Physiology through Physics, Comprising the Connexion of the Several Departments of Physical Science, Their Dependence on the Same Laws, and the Relation of the Material to the Immaterial

**Published:** 1844-04

**Authors:** 


### Art. XVII.

*The Sources of Physical Science; being an Introduction to the Study of Physiology through Physics, comprising the connexion of the several departments of Physical Science, their dependence on the same Laws, and the relation of the Material to the Immaterial.* By Alfred Smee, F.R.S., Surgeon to the Bank of England, &c.—*London*, 1843. 8vo, pp. 296.

Mr. Smee is well known to the scientific world, as the inventor of a valuable modification of the Voltaic battery, and as the improver of the electrotype process, which is now so widely introduced into the arts and manufactures,—mainly, we believe, through the development given to the original idea, in his ingenious and fertile mind. We had the pleasure, not long since, of noticing a second edition of his treatise on Electro-metallurgy; and the volume before us is a fresh evidence of the zeal and industry with which he pursues his scientific inquiries, in the midst of professional avocations, and we may say in connexion with them: for, as we learn from the preface, it is the first offspring of an attempt, in which the author has long been engaged, to "investigate the physical structure of man, and to endeavour to unravel the mysterious means by which all physical forces, when acting on the human frame, are converted into nervous impressions." Such an inquiry, conducted by an individual well versed in physical science, and with a competent knowledge of physiology, holds out much promise of valuable results. It is only when all the operations, for which the laws of physics can account, have been investigated and set aside, that we can distinguish the purely *vital* operations, the laws of which constitute the science of physiology; and the boundary between the two is very far from being distinctly marked out.

For the better examination of the sources, from which the various phenomena of physical science originate, Mr. Smee intended to draw up a slight sketch of the inquiry, to form an introductory chapter to his physiological investigations. But finding that it extended to a much greater length than he had anticipated, he determined to publish it as a separate treatise, in the hope, as he modestly expresses himself, that the compressed view of the subject which it contains, might possibly lead those who possess greater time and opportunity, to devote their attention more particularly to the essential nature and mutual relations of the various divisions of physical science. "If I shall hereafter find," he says, "that my labours have been useful to society, or have induced others to produce a more perfect treatise, I shall feel most amply rewarded."

Although nearly resembling in its title, the well-known 'Connexion of Physical Sciences' of Mrs. Somerville, the work of Mr. Smee essentially differs from it in plan and objects; his purpose being rather to exhibit the *fundamental* connexion between them, by showing that their varied phenomena are produced by agencies of the same character; whilst *hers* is rather to show the conjoint participation of several agencies in the phenomena of the respective sciences. If we might illustrate our meaning by a simile, we should say that the one aims to show the connexion of the branches into a common trunk, whilst the other demonstrates the interlacements of their ramifications.

We may best give an idea of the plan and scope of Mr. Smee's work, by the following summary, with which it concludes:

"Matter is matter, and solely exists by the will of God.

" Matter is made up of finite particles, or atoms; a series constituting number, and the study of number, arithmetic.

| | |
|---|---|
| Particles of matter *attracted together* give rise to . . . | Form,<br>Volume,<br>Composition,<br>Cohesion,<br>Adhesion,<br>Position. |
| Peculiarity in the *direction* of attractions produces . . | Crystallization,<br>Polarity,<br>Magnetism. |
| Attraction *acting on* attracted matter causes . . . | Tension, a tendency for action,<br>Force, a capacity for action |
| Force, by *destroying* the attractions of attracted matter, exhibits . . . . . . | Galvanic phenomena,<br>Electric ditto,<br>Electro-magnetic ditto,<br>Motion,<br>Disintegration,<br>Decomposition. |
| The results of force, in consequence of the *resistance* of old or previously-existing attractions, produce the phenomena called . . . | Time,<br>Heat,<br>Light,<br>Sound,<br>Odour. (?) |
| These latter, being the result of force, exhibit . | The effect of force generally, and therefore capacity for the destruction of attractions." |

Into the details, it is scarcely our province to enter; but having bestowed some time and attention on the perusal of the volume, we feel qualified to offer an opinion as to its merits. A large proportion of it consists of scientific *facts*, of which some are familar to every one, whilst others are less generally known; but they are all placed in new and frequently very striking positions, so as often to present themselves under an aspect entirely different from that in which we have been accustomed to regard them. Hence no one can attentively read the book without feeling a strong interest in it, and experiencing benefit from it. But we are bound to add, that the execution of the philosophical portion of the treatise is in our opinion inferior to its plan; and that, in his anxiety to avoid abstractions, the author has fallen, in many instances, into what appear to us very grave errors. We cannot think he is right, for example, in the adoption of *number*, as one of his three fundamentals, along with matter and attraction, (Preface, p. vii;) since it is merely the repetition of matter, and not anything distinct from it. Nor can we regard any philosophical system as correct, which introduces *time* into the same category with heat, light, &c., and classes it as a *material property*. We doubt if the way is yet clear for the kind of generalization at which Mr. Smee has aimed; and we think that, when the period does arrive, the advance will probably emanate from a mind more experienced in philosophic speculation,—that, for instance, of a Herschel or a Faraday,—than it is fair to expect Mr. Smee's at present to be. Nevertheless he has produced a work, which possesses many claims upon the attention of those who are interested in physical science, and which will serve, we trust, the purposes at which he has aimed. His own *forte* evidently lies in the *application* of principles, rather than in their development; and we expect much from his promised experimental investigations into the physical phenomena of life.

